# Lymph nodes may be a source for immunetherapy in gastric cancer

**DOI:** 10.18632/oncotarget.27578

**Published:** 2020-05-12

**Authors:** Paula Baraúna Assumpção, Erika Couto Canelas, Aline Cruz Ramos, Ana Anaissi, João Felipe Acioli, Geraldo Ishak, Sidney Santos, Samia Demachki, Paulo Assumpção

**Affiliations:** ^1^Laboratório Genética Humana e Médica, Universidade Federal do Pará, Belém-PA, Brasil; ^2^Núcleo de Pesquisas em Oncologia, Hospital Universitário João de Barros Barreto, Belém-PA, Brasil; ^3^Serviço de Cirurgia do Hospital Universitário João do Barros Barreto, Belém-PA, Brasil; ^4^Instituto de Ciências da Saúde, Universidade Federal do Pará, Belém-PA, Brasil

**Keywords:** gastric cancer, immunotherapy, lymph nodes

## Abstract

Background: adoptive immunotherapy is a promising cancer therapy. Immune cells are capable of recognizing and destroying cancer cells and represent a powerful strategy, however, this approach remains technically complicated, due to the need to select and isolate immune cells from these, present cancer antigens to those cells, expanding and reinjecting them. Lymph nodes recovered during gastric cancer surgery may represent an option for immunotherapy, since they harbor an enormous amount of immune cells, which have already been presented to cancer antigens. The advantage of selecting only cancer-negative lymph has not been determined yet. The status of immune checkpoints in the immune cells within the lymph nodes was analyzed in order to try to solve this problem.

Materials and Methods: Tissue microarrays were constructed and automated immunostaining for PD-1 and PD-L1 was performed on 143 lymph nodes from 70 patients with gastric adenocarcinoma.

Results: In positive nodes, PD-L1 was only positivity in cancer cells (6%) and PD-1 was positive for B lymphocytes (60%), T lymphocytes (70%) and one case in cancer cells (2.5%). In negative nodes, most cases were positive for PD-1 in B (73.1%) and T (71.65%) lymphocytes.

Conclusions: Expression of PD-1 and PD-L1 in gastric cancer lymph nodes was demonstrated for the first time. PD-1 is expressed in positive and negative nodes, which could activate the PD-1 pathway. Lymphocytes from tumor-free lymph nodes were negative for PD-L1, and this might represent an advantage for selecting these lymph nodes as a potential source of immune cells for adoptive immunotherapy.

## INTRODUCTION

Elements of the immune repertoire have gained increased attention since “avoidance of immune response” as one of the hallmarks of cancer [1] and introduction of immune check point therapy for diverse cancer types [2–5]. Nevertheless, the importance of preserving effectors of the immune system (such as lymphocytes in regional lymph nodes) that do not harbor cancer cells, seems to be under-valued.

Classical immune treatments, including adoptive therapy [6], have evolved to include the refined *ex-vivo* manipulation of diverse cells from the immune set, which has allowed innovative approaches both in clinical practice and as part of ongoing experimental and translational innovations [7–11]. The resection of regional lymph nodes in gastric cancer surgery is considered one of the most relevant advances, since it seems to be associated with benefits both in the reduction of local recurrence and probably in terms of survival [12–14].

The guidelines for gastric cancer lymphadenectomy [15, 16] include the systematic resection of lymph nodes regardless of whether they are positive or negative for cancer [17].

The enlargement of regional lymph nodes occurs due to the clonal expansion of lymphocytes that were exposed to immunogenic tumor cells, and as a consequence, became able to identify these tumor cells and eliminate them. These lymph nodes are probably not enemies that should definitely be discarded, but rather, they may be useful elements that can help control the disease. The large number of immune cells available inside the lymph nodes and the large number of retrieved lymph nodes might represent an optional source of immune cells that can be used for adoptive therapy. Moreover, the possibility for the selection of tumor-free lymph nodes, as will be discussed below, should represent an extra advantage that should be considered in the setting of adoptive immunotherapy.

### The use of lymphocytes from negative lymph nodes as ammunition against gastric cancer

Reflecting on the strategies that use tumor infiltrating lymphocytes (TILs) that are recovered from tumors and expanded *in vitro* and that are finally re-infused into patients, the aim is that these “adopted” cells might find and destroy tumor cells from where they were collected [6, 18]. Lymphocytes from lymph nodes could also exert the same functions, and additionally, might have the following advantages over TILs: lymphocytes are plentiful and easily available [19, 20]; if only negative lymph nodes are selected, undesirable effects of the tumor microenvironment would be avoided; lymph nodes are systematically resected during gastric cancer surgeries.

Unfortunately, in the war against gastric cancer, the “enemy” dominates the microenvironment in the great majority of battles. One of the most successful applied strategies in regard to tumor cells is the use of rogue messages to turn off immune system defenses. This is the case with PD-L1 and PD-L2 expression by tumor cells, as their expression activates PD-1 and results in the inhibition of the lymphocyte response; this leaves tumor cells undisturbed [21–25].

By using TILs collected from the tumor microenvironment, the strength of treatment might be partially repressed by the tumor strategy described herein, as should be the case when lymphocytes from positive lymph nodes are used. However, this would not be the case if the lymphocytes were selected from tumor-free lymph nodes, which would avoid the cancer strategy of silencing the immune response.

To determine if negative lymph nodes retrieved by a typical lymphadenectomy procedure performed during conventional surgical treatment for gastric cancer could be a cell source for adoptive immunotherapy, we analyzed the expression of PD-1 and PD-L1 in positive and negative lymph nodes.

## RESULTS

The results are expressed according to the immunostaining for PD-1 and PD-L1 in both immune cells and cancer cells and are reported in lymph nodes retrieved as part of lymphadenectomy during routine gastrectomies for adenocarcinomas.

The most of cases were men over 50 years of age who were treated at advanced disease stages; most cases were of the intestinal type according to the Lauren classification. The results of immunostaining for both PD-1 and PD-L1 among positive lymph nodes are shown according to histopathologic characteristics (Table 1).

**Table 1:** Immunostaining for PD-1 and PD-L1 in positive lymph nodes according to histopathologic characteristics

Histopathological Features		PD-L1			PD-1	
		Negative	Positive		Negative	Positive
*Histologic grade*	***n* = 30 (%) **			***n* = 40 (%) **		
*G1 (well differentiated)*	19 (63,3)	17 (56,6)	2 (6,7)	28 (70)	7 (17,5)	21 (52,5)
*G2 (Moderately differentiated)*	2 (6,7)	2 (6,7)	0	2 (5)	1 (2,5)	1 (2,5)
*G3 (Poorly differentiated)*	9 (30)	9 (30)	0	10 (25)	4 (10)	6 (15)
Histologic type						
*Intestinal*	19 (63,3)	17 (56,6)	2 (6,7)	28 (70)	7 (17,5)	21 (52,5)
*Diffuse*	9 (30)	9 (30)	0	10 (25)	4 (10)	6 (15)
*Mixed*	2 (6,7)	2 (6,7)	0	2 (5)	1 (2,5)	1 (2,5)
pT						
*T1 (early)*	1 (3,3)	1 (3,3)	0	2 (5)	0	2 (5)
*T2, T3 e T4 (Advanced)*	29 (96,7)	27 (90)	2 (6,7)	37 (92,5)	12 (30)	25 (62,5)
*Not reported*	0	0	0	1 (2,5)	0	1 (2,5)
Pn						
*N1*	4 (13,3)	4 (13,3)	0	6 (15)	1 (2,5)	5 (12,5)
*N2*	12 (40)	12 (40)	0	14 (35)	3 (7,5)	11 (27,5)
*N3*	14 (46,7)	12 (40)	2 (6,7)	20 (50)	8 (20)	12 (30)
pM						
*Mx*	*28 (93,3)*	26 (86,6)	2 (6,7)	37 (92,5)	10 (25)	27 (67,5)
*M1*	*2* (6,7)	2 (6,7)	0	*3 (7,5)*	2 (5)	1 (2,5)

PD-L1 was related to well differentiated tumors, of intestinal type, in advanced stages with positive lymph nodes. While PD-1 positive cases included both low and high grade tumors, either in early and advanced stages, mainly from intestinal type and with positive lymph nodes.

**Figure 1:**
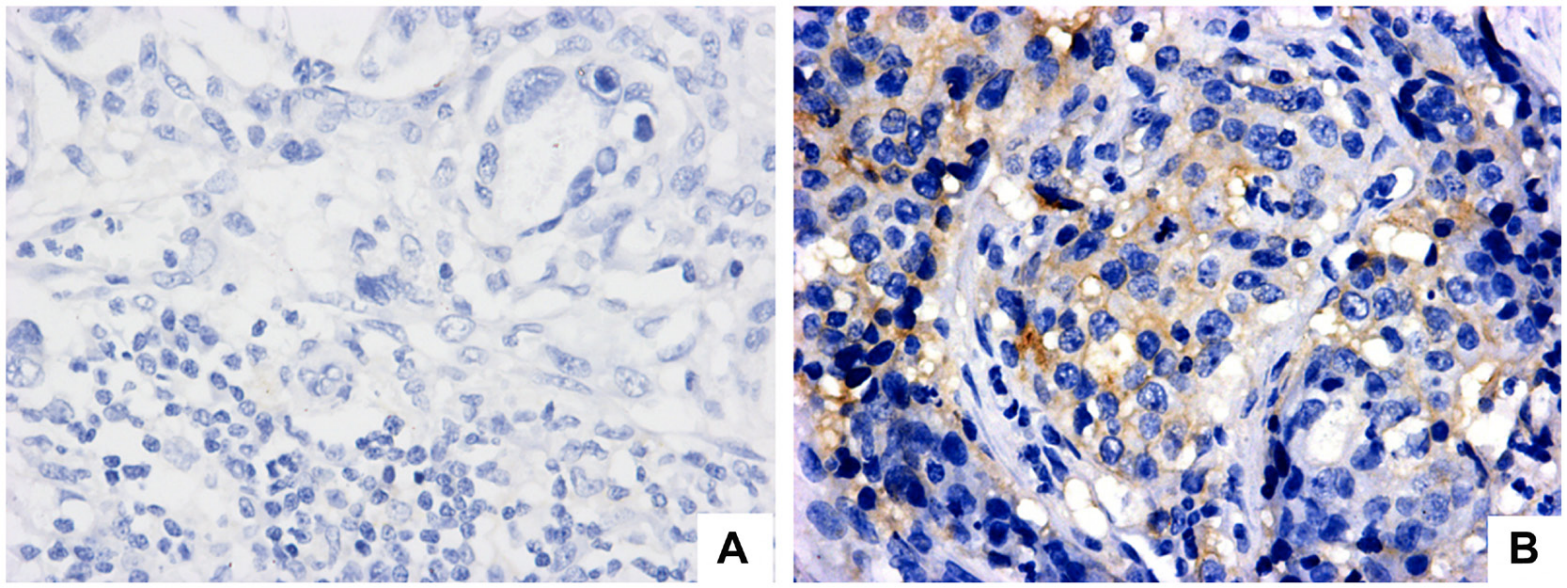
PD-L1 expression evaluation in regional lymph node with metastatic gastric adenocarcinoma (positive lymph node). (**A**) Negative staining in neoplastic cells (magnification 40×). (**B**) PD-L1 positive cytoplasmatic staining in neoplastic cells (magnification 40×).

PD-L1 positivity in cancer cells was observed in two cases (6%) (Figure 1B), which were both from advanced-stage disease, and were of the intestinal type; no staining was observed in immune cells (Figure 1A). In regard to PD-1, among immune cells, 24 cases had positive B lymphocytes (60%), while 28 cases had positive T lymphocytes (70%); staining was negative in all macrophages. Only one case presented PD-1 positivity in cancer cells (2.5%) (Table 2).

**Table 2:** Immunostaining for PD-1 and PD-L1 in B lymphocytes, T lymphocytes, macrophages and neoplastic cells from positive lymph nodes

Cell Type	PD-L1	PD-1
	*n* = 30 (%)	*n* = 40 (%)
**B Lymphocytes**		
NEGATIVE (< 1%)	30 (100)	16 (40)
> 1–5%	0	0
> 5–10%	0	15 (37,5)
> 10–25%	0	9 (22,5)
**T lymphocytes**		
NEGATIVE < 1%	30 (100)	12 (30)
POSITIVE > 1–5%	0	28 (70)
**Macrophages**		
NEGATIVE < 1%	30 (100)	40 (100)
POSITIVE > 1–5%	0	0
**Neoplastic cells**		
NEGATIVE < 1%	28 (94)	39 (97,5)
POSITIVE > 1–5%	2 (6)	1 (2,5)

In terms of the negative lymph nodes, the immunostaining for both PD-1 and PD-L1 show the follow distribution according histopathologic characteristics (Table 3).

**Table 3:** Immunostaining for PD-1 and PD-L1 in negative lymph nodes according to histopathologic characteristics

Histopathological Features		PD-L1			PD-1	
		Negative	Positive		Negative	Positive
*Histologic grade*	***n* = 68 (%) **			***n* = 67 (%) **		
*G1 (well differentiated)*	48 (70,6)	48 (70,6)	0	48 (61,6)	13 (19,4)	35 (52,2
*G2 (Moderately differentiated)*	2 (2,94)	2 (2,94)	0	2 (3)	0	2 (3)
*G3 (Poorly differentiated)*	18 (26,5)	18 (26,5)	0	17 (25,4)	3 (4,5)	14 (20,9)
Histological type						
*Intestinal*	48 (70,6)	48 (70,6)	0	48 (61,6)	13 (19,4)	35 (52,2)
*Diffuse*	18 (26,5)	18 (26,5)	0	17 (25,4)	3 (4,5)	14 (20,9)
*Mixed*	2 (2,94)	2 (2,94)	0	2 (3)	0	2 (3)
pT						
*T1 (early)*	10 (14,7)	10 (14,7)	0	10 (14,9)	2 (3)	8 (11,9)
*T2, T3 e T4 (Advanced)*	53 (77,9)	53 (77,9)	0	53 (79,1)	12 (17,9)	41 (61,2)
*Not reported*	5 (7,35)	5 (7,35)	0	4 (6)	2 (3)	2 (3)
pN						
*N0*	14 (20,6)	14 (20,6)	0	14 (20,9)	4 (6)	10 (14,9)
*N1*	13 (19,1)	13 (19,1)	0	13 (19,4)	2 (3)	11 (16,4)
*N2*	16 (23,5)	16 (23,5)	0	16 (23,9)	3 (4,5)	13 (19,4)
*N3*	20 (29,4)	20 (29,4)	0	21 (31,3)	6 (8,9)	15 (22,4)
*N4*	1 (1,5)	1 (1,5)	0	0	0	0
*Not reported*	4 (5,9)	4 (5,9)	0	3 (4,5)	1 (1,5)	2 (3)
pM						
*Mx*	66 (97)	66 (97)	0	64 (95,5)	15 (22,4)	49 (73,1)
*M1*	2 (3)	2 (3)	0	3 (4,5)	1 (1,5)	2 (3)

PD-1 positive cases included both low and high grade tumors, either in early and advanced stages, mainly from intestinal type and with positive lymph nodes. PD-L1 was negative since there weren’t cancer cells.

Three cases demonstrated PD-L1 positivity in macrophages, but none demonstrated positivity in lymphocytes, and obviously, none demonstrated positivity in cancer cells (Table 4).

**Table 4:** Immunostaining for PD-1 and PD-L1 in B lymphocytes, T lymphocytes, and macrophages from negative lymph nodes

Cell Type	PD-L1	PD-1
	*n = 68 (%)*	*n = 67 (%)*
**B Lymphocytes**		
NEGATIVE (< 1%)	68 (100)	18 (26,9)
> 1–5%	0	27 (40,3)
> 5–10%	0	19 (28,35)
> 10–25%	0	3 (4,5)
**T lymphocytes**		
NEGATIVE < 1%	68 (100)	19 (28,35)
POSITIVE > 1–5%	0	48 (71,65)
**Macrophages**		
NEGATIVE < 1%	65 (95,6)	65 (97)
POSITIVE > 1–5%	3 (4,4)	2 (3)

**Figure 2:**
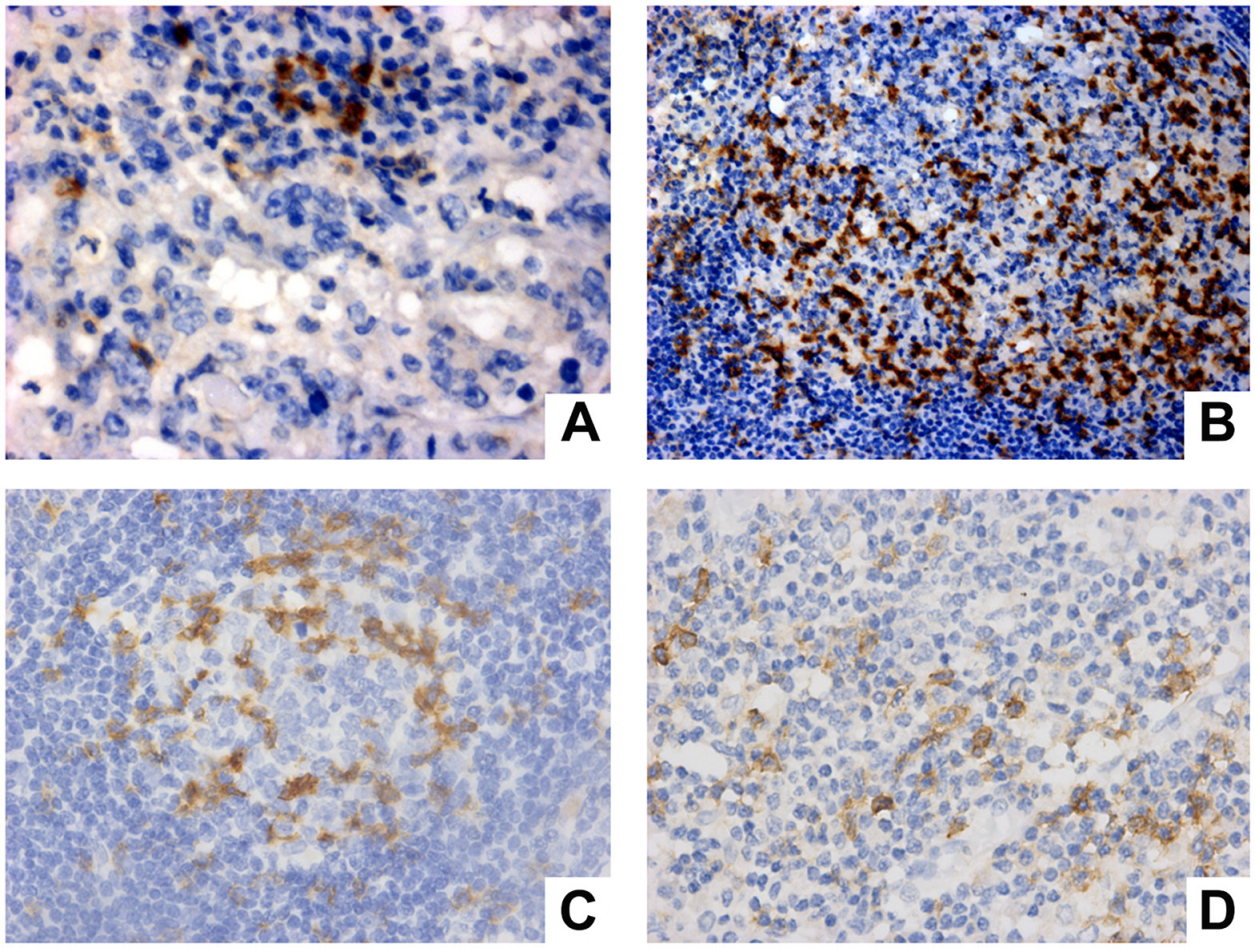
PD-1 expression evaluation in immune cells from positive lymph node (**A**, **B**) and negative lymph node (**C**, **D**). (A) PD-1 expression in T lymphocytes (Magnification 40×). (B) expression in centrofollicular B lymphocytes (Magnification 20×). (C) PD-1 expression in centrofollicular B lymphocytes of negative lymph node (Magnification 40×). (D) PD-1 expression in macrophage and T lymphocytes (magnification 40×).

These results are represented in Figure 2. On the contrary, the majority of cases presented PD-1 positivity both in B (73.1%) and T (71.65%) lymphocytes (Figure 2A and 2C). In addition, two cases presented PD-1 positivity in macrophages (Figure 2B and 2D).

## DISCUSSION

Adoptive immunotherapy has shown promising results in individuals with different cancer types [26, 27], and in terms of gastric cancer treatment, this therapy has also seemed to improve survival [28]. Nevertheless, due to new concepts in immunotherapy and cancer, the microenvironment has gained relevance and has emerged as a necessary concomitant target to consider while selecting cancer immunotherapy strategies [29, 30].

Cancer cells appear to control the tumor microenvironment, including the avoidance of local cells of the immune system [1] via the inhibition of the host response against the disease. Therefore, we realized that the selection and isolation of T cells and even other immune cells that are able to recognize and eliminate tumor cells, but that are not subjugated by cancer cells with the power to silence the immune response, might represent a potential approach to be investigated in gastric cancer immunotherapy.

Gastrectomy with lymphadenectomy is the standard surgical procedure for the treatment of gastric cancer [15, 16]. The systematic resection of lymph nodes provides a large number of both negative and positive lymph nodes, depending on the stage of the disease. The large number of lymphocytes and/or other immune cells retrieved from the negative lymph nodes constitutes a source of possible “bullets” to be selected, saved, and improved, with the aim to allow efficient and targeted adoptive immunotherapy.

The presence of cancer cells in the lymph nodes, which are considered classical “positive nodes,” implies the possibility of an ongoing cancer cell strategy in which cancer cells escape the immune response. This was previously demonstrated by the activation of immune check points as a consequence of tumor cell expression of PD-L1 and other molecules in the tumor microenvironment.

The results presented in this preliminary investigation are original since the only published data regarding PD-1 and PD-L1 expression in gastric cancer are related to either tumors [31] or non-cancer gastric mucosae [32]. The results from the current study also corroborate the hypothesis that negative lymph nodes seem to be free of the inhibition of the immune response, which is frequently found in the presence of malignant cells.

Although PD-1 expression was observed in the majority of both positive and negative lymph nodes, the activation of the PD-1 pathway depends on the concomitant expression of its ligand [24]. Since PD-L1 was not expressed in lymphocytes from negative nodes, even in those that expressed PD-1, the inhibition of the immune response by a PD1/PDL1 interaction would never occur, if these lymph nodes were the only ones selected for adoptive immunotherapy. In contrast, the expression of PD-L1 in “positive nodes” might inhibit the immune response by activation of the PD-1 pathway.

This strengthens the hypothesis of a possible negative influence of using lymphocytes exposed to tumor cells such as TILs or even lymphocytes from positive nodes. Obviously, after the administration of immune cells that have been expanded *in vitro*, the expression of PD-1 ligands and/or activation of other tumor mechanisms for avoiding the host immune system might protect some cancer cells from immune system attack and subsequent destruction.

As is the case in most wars, the enemies try to eliminate each other and protect themselves while in inconvenient environments. Nevertheless, the criteria for the selection of combatants to send to the battlefield favors the strongest and not ones who are easily subdued by the enemy, even though some might fail at their responsibilities.

The silencing of adoptive immune cells after infusion might occur independently of using cells derived from tumors or lymph nodes and might be circumvented by the concomitant use of immune check point antibodies, if required.

Additionally, PD-1 expression in selected lymphocytes could be definitively silenced *in vitro* by recently available techniques such as DNA editing [33], like CRISPR [34], which would thus avoid the activation of the PD-1 pathway after re-infusion of the lymphocytes, even in cases of tumors that express PD-1 ligands.

Nevertheless, it seems to make sense to select immune cells that are easily available from routinely retrieved lymph nodes and ones that are not exposed to silencing cues (PD-1 ligands and others), rather than collecting them from the tumor mass.

The small number of PD-L1-positive cells in this casuistry is in accordance with the expected positive expression of this binding protein in primary gastric cancers, since it seems to be related to the EBV molecular subtype, which is the least common among the gastric cancer subtypes [35].

The finding of PD-L1 expression in macrophages was previously reported [36] and might be related to the immune function of these cells, which phagocyte cancer cells and other cell types, and whose aim is to clear the microenvironment of harmful molecules that threaten tissue homeostasis.

Although modest, these preliminary results could be extrapolated to investigations of many other tumors, such as colon cancer, breast cancer and ovarian cancer, for which lymphadenectomy is also applied.

In conclusion, the expression of PD-1 and PD-L1 in lymph nodes retrieved during gastrectomies was demonstrated here for the first time. These proteins are expressed in lymphocytes from lymph nodes, but PD-L1 is not expressed in lymphocytes from negative lymph nodes. Negative for malignancy lymph nodes retrieved during gastrectomies might be considered a potential source for gastric cancer immunotherapy.

## MATERIALS AND METHODS

This study was approved by the Universidade Federal do Pará Ethics Committee with CAAE N° 43961215.9.0000.5634, all research was performed in accordance with relevant guidelines/regulations and all participants read and signed the written informed consent. One hundred and forty-three lymph nodes from 70 gastric adenocarcinoma patients who submitted to curative intended gastrectomy with D2 classical lymphadenectomies were selected for the analysis. The surgical specimens were evaluated by the same pathologist, and tumors were classified according to the Lauren and WHO standards, and according to the 8th edition of the UICC staging system.

Lymph nodes were initially assessed by conventional microscopy and were classified as positive (with metastasis) or negative (without metastasis). A total of 69 positive lymph nodes and 74 negative lymph nodes were initially stained and were analyzed by microscopy, after which, at least two, representative samples were selected from each node.

### Tissue microarrays (TMAs)

Histological sections from each tissue block were stained with Hematoxylin and Eosin (H&E) and were carefully reviewed by a pathologist. The most representative areas of each lymph node were selected for analysis. Cylindrical cores from the selected areas were removed to construct TMA blocks using TMA Grand Master (3DHISTECH Ltd.) according to the manufacturer’s instructions.

### Immunohistochemistry

The immunohistochemistry procedures were performed at Gx Ventana^®^ (Ventana Medical Systems, Inc. Tucson, AZ, USA) with an Ultra View Universal Dab Detection Kit. Briefly, 3-μm-thick sections were obtained from the TMA blocks, and immunohistochemistry was performed using a rabbit polyclonal PDL-1 antibody (Abcam^®^, ab205921, 28-8, RabMab) at a 1:200 dilution and a mouse monoclonal PD-1 antibody (Abcam^®^, ab52587, NAT-105) at a 1:50 dilution. Both tumour cells and immune cells, such as B and T lymphocytes and macrophages, were analyzed. The criteria for positive PD-L1 protein staining in tumor cells was restricted to cytoplasmic membrane staining, either partially or completely, while for immune cells, membrane or cytoplasmic staining for both PD-L1and PD-1 was considered positive [37].

In some cases, although the TMAs contained two samples from each case, due to technical problems, the analyses failed for one or both antibodies. These failures primarily include disruption of the samples, following performing the transfer of the slices from the receptor blocks during construction of the TMA slides for staining with the selected antibodies.
